# Molecular epidemiological study of *Trichomonas gallinae* focusing on central and southeastern Europe

**DOI:** 10.3389/fvets.2022.1050561

**Published:** 2022-12-15

**Authors:** Barbara Tuska-Szalay, Gábor Sipos, Nóra Takács, Jenő Kontschán, Attila D. Sándor, Áron Péter, Krisztián Berta, Ádám Kerek, Ákos Jerzsele, Jan Votýpka, Sándor Hornok

**Affiliations:** ^1^Department of Parasitology and Zoology, University of Veterinary Medicine, Budapest, Hungary; ^2^ELKH-ÁTE Climate Change: New Blood-Sucking Parasites and Vector-Borne Pathogens Research Group, Budapest, Hungary; ^3^Centre for Agricultural Research, Plant Protection Institute, ELKH, Budapest, Hungary; ^4^Department of Parasitology and Parasitic Diseases, University of Agricultural Sciences and Veterinary Medicine, Cluj-Napoca, Romania; ^5^Szent Bernát Small Animal Ambulance, Budapest, Hungary; ^6^Department of Pharmacology and Toxicology, University of Veterinary Medicine, Budapest, Hungary; ^7^Biology Centre, Institute of Parasitology, Czech Academy of Sciences, České Budějovice, Czechia; ^8^Department of Parasitology, Faculty of Science, Charles University, Prague, Czechia

**Keywords:** Trichomonadea, 18S rRNA gene, alpha-tubulin gene, Columbiformes, pigeon, dove

## Abstract

*Trichomonas gallinae* is a geographically widespread protozoan parasite of birds. In this study, oropharyngeal swab samples were collected in Hungary and Romania from 99 columbiform birds, including 76 feral pigeons (*Columba livia domestica*: 42 kept for racing, 32 with urban and two with rural habitat), four common wood pigeons (*C. palumbus*), 16 ring doves (*Streptopelia risoria*) and three Eurasian collared doves (*S. decaocto*). These samples were analyzed for the presence of *T. gallinae* using molecular methods. Racing feral pigeons had significantly higher prevalence of *T. gallinae* infection than urban feral pigeons. The rate of PCR-positivity was the highest among wood pigeons and ring doves. Based on 18S rRNA gene, *T. gallinae* was the most heterogenous among racing feral pigeons sampled in a trading-breeding place. Clinical signs were associated with only one 18S rRNA gene subtype. The most divergent 18S rRNA gene subtype, *Trichomonas* sp. Hu-TG37 clustered with *T. canistomae* and *T. tenax* and represents probably a new species. To our knowledge, this is the first report on the genetic diversity of *T. gallinae* in the southeastern European region. The results suggest that most detected *T. gallinae* 18S rRNA gene subtypes are not host-specific and do not cause clinical signs. The highest number of 18S rRNA gene subtypes was demonstrated among racing feral pigeons. Significantly more captive than free-living columbiform birds had *T. gallinae* infection. These data highlight the importance of epizootic monitoring of the genetic diversity and presence of *T. gallinae* in trading-breeding places of pigeons and doves.

## Introduction

*Trichomonas gallinae* (Parabasalia: Trichomonadida) is a widespread flagellated protozoan parasite of birds from various orders, including Columbiformes, Accipitriformes, Strigiformes, Psittaciformes, Falconiformes and Passeriformes (1–3). Among columbiform birds, the rock pigeon (*Columba livia*) is the main reservoir of this parasite. The most important route of trophozoite transmission between birds is oral by saliva, through shared water and food sources (2, 4, 5). In columbids the predominant way of spreading to nestlings is *via* crop milk (1). Additionally, predatory birds can become infected by consuming a carrier prey item, since trichomonas trophozoites may survive in carcasses for at least 48 h (2, 4, 5). *Trichomonas gallinae* can persist for up to 1 h in various water sources, e.g., in gutters and drinkers (6), but higher temperatures (30–35°C) can further prolong its survival (7). Although it is able to form pseudocyst in unfavorable conditions, the moist environment is essential to maintain its infectivity (2).

*Trichomonas gallinae* is often considered a normal inhabitant (commensal) of the mucosal surface in the upper gastrointestinal tract (2). However, by eliciting inflammation in the underlying tissues or when entering more distally the digestive tract of birds, this protozoan parasite might cause mild to severe lesions depending on strain virulence and host susceptibility. Infection with highly pathogenic strains may lead to death. However, columbiform birds may also be asymptomatic carriers of *T. gallinae*, ensuring the carefree spread of this protozoon. In addition, if protective immunity develops, affected birds become resistant to a new infection (2, 4, 8, 9).

Regarding pathogenesis, *T. gallinae* trophozoites establish preferentially in the upper gastrointestinal tract (the oropharynx, esophagus and crop), where they can cause yellowish necrotic lesions. In severe cases trichomonosis can lead to starvation and suffocation. Furthermore, *T. gallinae* can spread to tissues of the cranium, thorax, and abdomen, as well as of the liver and air sacs causing similar lesions, with deepening tissue involvement, referred to as canker (1). Recently, massive death of passeriform birds (the greenfinch, *Chloris chloris*, and the goldfinch, *Carduelis carduelis*) due to infection with a specific lineage of *T. gallinae* has been reported throughout Europe (3).

Avian trichomonosis has been reported to have a worldwide occurrence (2). In Europe, as on other continents, columbiform birds play the most significant role in the maintenance of *T. gallinae* (2). The prevalence in western and southern Europe is high among wild columbids (74%) (9), unlike in northern central Europe where only a little more than one third of racing pigeons proved to be PCR positive (10).

The main objective of this study was to investigate the prevalence, genetic diversity and phylogenetic relationships of avian *Trichomonas* species in Hungary and Romania, where no similar data are available. Sampled hosts included racing and urban feral pigeons as well as other highly urbanized or pet columbiform bird species (Eurasian collared doves: *Streptopelia decaocto*, common wood pigeons: *C. palumbus* and ring doves: *S. risoria*, respectively). The nomenclature used in the context of feral pigeons complies with genetic and ornithological studies on this species (11, 12).

## Methods

### Sample collection

Oropharyngeal mucosal samples were collected with sterile cotton swab applicators randomly from 99 columbiform birds that underwent routine veterinary examination in Hungary (*n* = 77) and Romania (*n* = 22) between May and August, 2021. Four avian host species were sampled, each bird on one occasion, including feral pigeons (*Columba livia domestica*: 42 racing, 32 urban and 2 rural), wood pigeons (*n* = 4), ring doves (*n* = 16) and Eurasian collared doves (*n* = 3) (Supplementary Table 1). Racing pigeons were sampled at a trading-breeding place in Csepel (Budapest, Hungary). To assess the necessity of culturing *T. gallinae* prior to DNA extraction, the swab sampling was performed in duplicates from 20 racing pigeons, and one swab sample was placed into 8 ml CPLM culture medium with *Trichomonas* selective supplement (Biolab Diagnostics Laboratory Inc., Budapest Hungary), containing streptomycin, penicillin and sterile inactivated horse serum (pH adjusted to 6). These cultures were kept at 37°C for 2 days. All other swab samples were placed in 2 ml sterile Sarstedt tubes and frozen at −20°C.

### DNA extraction and PCR methods

DNA was extracted with the QIAamp DNA Mini Kit (Qiagen, Hilden Germany) according to the manufacturer's blood or tissue protocol, with slight modification. In particular, DNA extraction was performed from 200 μl of culture medium in duplicates, after adding 200 μl AL buffer and continuing with the blood DNA extraction protocol. On the other hand, thawed swabs were overlaid with 200 μl AL buffer and 200 μl sterile PBS, incubated for 10 min at 56°C prior to removal of cotton swab from the fluid, followed by adding proteinase-K and continuing the procedure according to the tissue protocol. In each group of 23 samples an extraction control (180 μl tissue lysis buffer) was included to monitor cross-contamination.

All DNA extracts and extraction controls were analyzed with three conventional PCRs: first with a screening assay amplifying a short, approx. 500-bp-long fragment of the 18S rRNA gene to detect the presence of Trichomonadea, followed by a primary and a secondary assay for sequencing approx. 1,550–1,600 and 1,200-bp-long parts of two genetic markers (18S rRNA gene and alpha-tubulin genes, respectively). The reasons for selecting these two genetic markers were to include a conserved gene (18S rRNA) that is widely used in molecular characterization of *T. gallinae*, and a protein encoding gene (alpha-tubulin) for which sequences corresponding to 18S rRNA gene subtypes are available from North America (8) but not from Europe. The suitability of the screening assay was checked by sequencing PCR products of 13 samples which verified the presence of *T. gallinae* in all cases. Primers and cycling conditions of PCRs are summarized in the Technical Appendix. In these PCRs 5 μl of extracted DNA was added to 20 μl of reaction mixture containing 1.0 U HotStar Taq Plus DNA Polymerase (5 U/μl) (Qiagen, Hilden, Germany), 0.5 μl dNTP Mix (10 mM), 0.5 μl of each primer (50 μM), 2.5 μl of 10× Coral Load PCR buffer (15 mM MgCl_2_ included), 1 μl extra MgCl_2_ (25 mM) and 14.8 μl distilled water. Except for alpha-tubulin PCR where 15.8 μl distilled water was added without extra MgCl_2._ Sequence-verified *T. vaginalis* served as a positive control.

### Sequencing and phylogenetic analyses

The purification and sequencing of the PCR products were performed at Biomi Ltd. (Gödöllő, Hungary). The newly generated sequences were submitted to GenBank under accession numbers ON631556-ON631566 (18S rRNA gene, long fragment) and ON808545-ON808550 (alpha-tubulin gene) (Supplementary Table 1). The 18S rRNA gene subtypes are designated as A to E here independently from other studies, for instance where ITS was used for this purpose (13).

Obtained sequences were compared to GenBank data using the nucleotide BLASTn program (https://blast.ncbi.nlm.nih.gov). All sequences retrieved from GenBank and included in the phylogenetic analysis had nearly or exactly 100% coverage with sequences from this study. The dataset was resampled 1,000 times to generate bootstrap values. Phylogenetic analysis was conducted by using the maximum-likelihood method and the Jukes-Cantor model according to the best-fit selection with the program MEGA 7.0 (14). Prevalence data were compared with Fisher exact test. Differences were regarded significant when *P* < 0.05.

## Results

According to the preliminary comparison on the efficacy of molecular detection of *T. gallinae*, 19 out of 20 swab samples but only 18 out of 20 culture medium samples were PCR positive (i.e., one pigeon was positive only by its culture medium sample vs. two pigeons diagnosed as infected only from their swab samples) (Supplementary Table 1: 7th column, samples TG1-50). Therefore, swab samples were used in the remaining part of the study.

*Trichomonas gallinae* was detected in all four studied bird species, with an overall prevalence of 73% (72 out of 99) (Table 1). Based on the screening assay, racing feral pigeons had a significantly (*P* < 0.0001) higher prevalence of *T. gallinae* infection (95%: 40 of 42) than urban feral pigeons (34%: 11 of 32). Among other columbiform bird species, the rate of PCR-positivity was the highest, 100% among wood pigeons (4 out of 4), followed in decreasing order by ring doves (94%: 15 out of 16) and collared doves (33%: 1 out of 3) (Table 1; Supplementary Table 1). These results also imply that the association of *T. gallinae* infection with artificially bred, captive columbiform birds (racing feral pigeons and ring doves) was highly significant (*P* < 0.0001) in comparison with free-living columbiform birds (urban and rural feral pigeons, wood pigeons and collared doves) (55/58 vs. 17/41, respectively).

**Table 1:** Results of molecular analyses for the 18S rRNA gene of Trichomonadea according to sample types of columbiform birds.

**Species or type of sample source**	**Country of origin**	Prevalence^**^ (positive/all)	**Long 18S rRNA gene subtype (*n*)**	**GenBank accession number**
Racing feral pigeon (*Columba livia*)	Hungary	97% (29/30)	**B** (16), **C** (3), *Trichomonas* sp. Hu-TG37 (1)	ON631556, ON631557, ON631566
	Germany^*^	89% (8/9)	**D** (3)	ON631558
	Denmark^*^	100% (2/2)	**B** (1)	ON631556
	Belgium^*^	–(1/1)	**B** (1)	ON631556
Urban feral pigeon (*Columba livia*)	Hungary	40% (4/10)	**B** (1), **D** (1)	ON631559, ON631560
	Romania	32% (7/22)	**A** (1), **D** (2)	ON631561, ON631562
Rural feral pigeon (*Columba livia*)	Hungary	50% (1/2)	–	–
Common wood pigeon (*Columba palumbus*)	Hungary	100% (4/4)	**A** (1)	ON631563
Ring dove (*Streptopelia risoria*)	Hungary	94% (15/16)	**B** (4), **E** (2)	ON631564, ON631565
Eurasian collared dove (*Streptopelia decaocto*)	Hungary	33% (1/3)	–	–

* Kept separately after arrival, but contact with Hungarian birds cannot be excluded;

** based on the short 18S rRNA gene screening PCR.

Based on the long fragment of the 18S rRNA gene, six *Trichomonas* subtypes were detected in 37 columbiform birds (Table 1). Compared to a reference sequence from North America (GenBank: EU215373), these had four to eight nucleotide differences (Table 2), i.e., 99.5–99.7% (1,471–1,475/1,479 bp) identity. In a smaller geographical context, the 18S rRNA gene sequences obtained in this study from central and southeastern Europe had higher, up to 11 nucleotide differences with conspecific sequences from western and southwestern Europe: only 99.3–99.7% (1,468–1,474/1,479 bp) sequence identity with *T. gallinae* from passeriform birds sampled in 2019 in France (e.g., MK172846), whereas 99.5–100% (1,443/1,450–1,449/1,449 bp) sequence identity with samples collected from columbiform birds in 2019 in Portugal (e.g., MK932772).

**Table 2:** Site-specific variations among 18S rRNA gene subtypes of *Trichomonas gallinae* and *Trichomonas* sp. Hu-TG37 compared to positions in EU215373 used as a reference sequence (identical with genotype A).

**18S rRNA**	**Position (in which the nucleotide is indicated below)**														
**gene subtype**	**33**	**215**	**384**	**386**	**408**	**418**	**590**	**592**	**648**	**825**	**837**	**850**	**1120**	**1143**	**1281**
**A**	A	T	T	A	G	C	C	A	C	A	G	T	A	A	C
**B**	–	–	–	T	–	–	–	–	–	–	A	–	C	G	–
**C**	–	–	–	T	–	–	–	–	–	G	A	C	C	G	–
**D**	–	–	A	T	–	–	T	T	T	–	–	–	C	G	–
**E**	–	–	–	T	–	–	–	–	T	G	A	C	C	G	–
**Hu-TG37**	T	A	–	T	A	T	–	–	–	–	–	–	T	T	T

Nucleotides that are identical to the reference are indicated with “–”. Note that only the last three positions shown in this table are covered by the screening (short 18S rRNA gene) PCR, i.e., genotyping was possible from 37 of 72 *Trichomonas*-positive specimens yielding sequenceable product in the long 18S rRNA PCR.

Only two genetic variants were detected among sympatric urban feral pigeons (subtypes B, D in Hungary; A, D in Romania), while four subtypes (B, C, D and the most divergent Hu-TG37) occurred in racing feral pigeons kept in one trading-breeding place (Table 1). Among racing feral pigeons, 18S rRNA gene subtype D was only found in birds that originated from Germany, while in Hungary subtypes A and E were exclusively found in wood pigeons and ring doves, respectively (Table 1). In the latter species, the occurrence of 18S rRNA gene subtypes was related to the origin (breeding place) of captive birds: subtype E was detected in two ring doves of one sampling locality, whereas subtype B in four birds at the other sampling locality (Supplementary Table 1). Clinical signs (lesions in the oropharyngeal cavity or the eyes, conjunctiva) were only associated with subtype D (Supplementary Table 1; Supplementary Figure 1).

Amplification and sequencing of part of the alpha-tubulin gene were successful from at least one sample representing each 18S subtype. Alpha-tubulin gene sequences obtained in this study had a lower (98.5–98.6%: 1,008–1,009/1,023 bp) or a higher level (99.6–99.7%: 1,019–1,020/1,023 bp) of sequence identity to that of an isolate (EU215382) used as a reference. Based on the corresponding amino acid sequences, most mutations in this protein encoding gene were synonymous, but subtypes E and D had a single amino acid difference compared to the reference isolate (valine instead of alanine at position 149, and isoleucine instead of valine at position 34, respectively).

**Figure 1:**
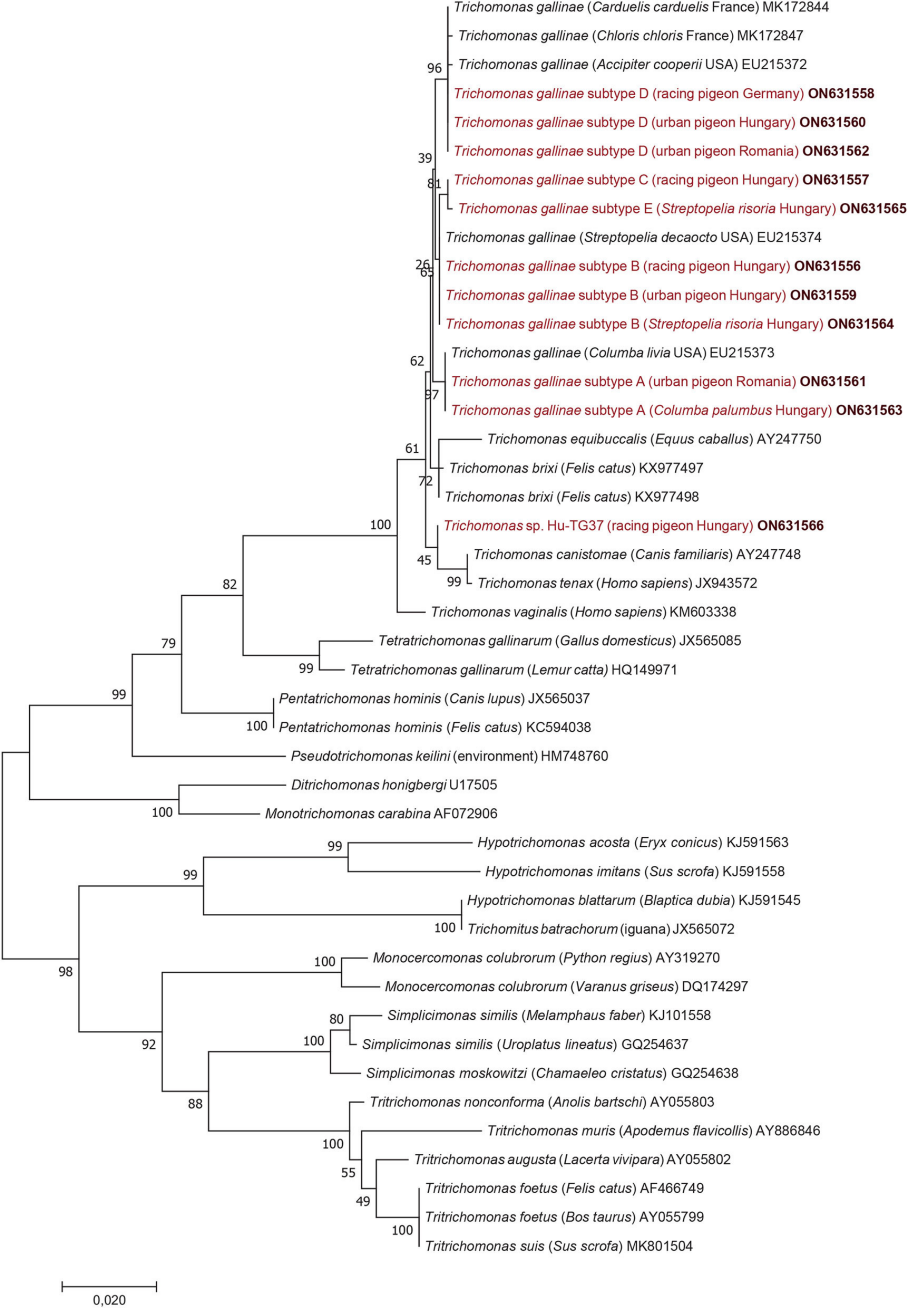
Phylogenetic tree of Trichomonadea based on the 18S rRNA gene, made with the maximum likelihood method and the Jukes-Cantor model. In each row, after the species or genus name, the isolation source, for *Trichomonas gallinae* the country of origin and the GenBank accession number are shown. Sequences obtained in this study and representing each subtype are in red and bold accession numbers. The analysis involved 44 nucleotide sequences and 1,000 bootstrap replications. There were a total of 1,121 positions in the final dataset. The scale-bar indicates the number of substitutions per site.

**Figure 2:**
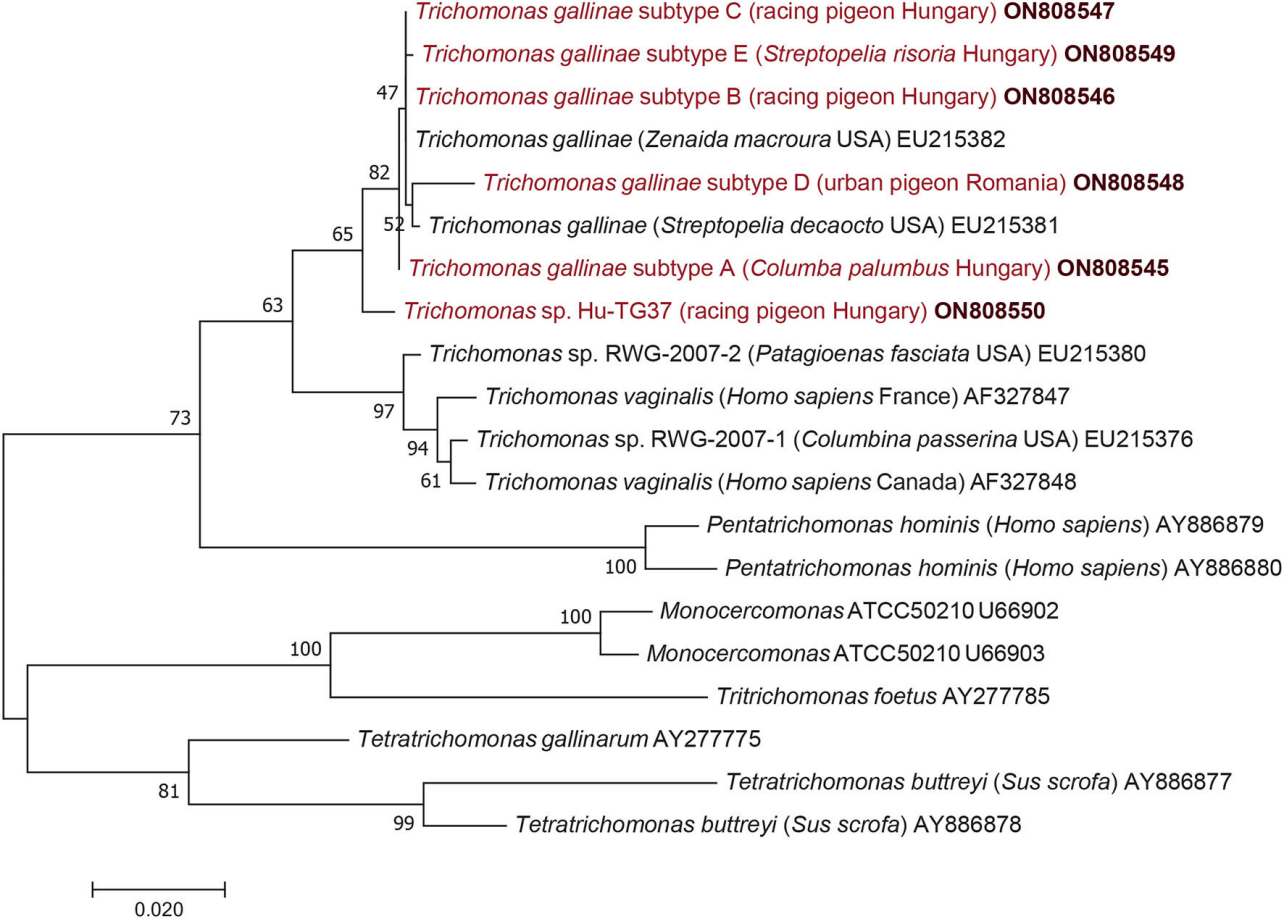
Phylogenetic tree of Trichomonadea based on the alpha-tubulin gene, made with the maximum likelihood method and the Jukes-Cantor model. In each row, after the species or genus name, the isolation source, for species closely related to *Trichomonas gallinae* the country of origin and the GenBank accession number are shown. Sequences obtained in this study and representing each subtype are in red and bold accession numbers. The analysis involved 20 nucleotide sequences and 1,000 bootstrap replications. There were a total of 977 positions in the final dataset. The scale-bar indicates the number of substitutions per site.

Considering the results of phylogenetic analyses, the topology of the 18S rRNA phylogenetic tree (Figure 1) did not show clear clustering of *T. gallinae* subtypes obtained in this study according to host species, living place (or keeping modes) of columbiform birds. However, subtype A (identified both in Hungary and Romania) formed a sister group to all other 18S rRNA gene subtypes (B, C, D, E): although with only moderate (65%) support (Figure 1) but confirmed by the alpha-tubulin phylogenetic tree (Figure 2). More importantly, *Trichomonas* sp. Hu-TG37 (detected in a racing feral pigeon from south Hungary) belonged to the phylogenetic group of *T. canistomae* and *T. tenax* with moderate (61%) support, implying, that this is a separate species (in other words: if this isolate would belong to *T. gallinae*, this species would not be monophyletic). The separation of *Trichomonas* sp. Hu-TG37 from *T. gallinae* was also confirmed by the alpha-tubulin phylogenetic tree (Figure 2).

## Discussion

To our knowledge, this is the first study on the genetic diversity of *T. gallinae* in Hungary, Romania and the whole southeastern European region, complementing previous reports from western, central and southern Europe (see below). Most infected birds in this study did not show clinical signs of trichomonosis except five pigeons (6.9%). A lower prevalence of clinical trichomonosis (0.37%) has been reported in a study involving 612 wild and domestic pigeons (13). Since *T. gallinae* can cause the death of infected hosts (15), the rarity of symptoms can in part be explained by the death of severely affected birds, which can die before they are examined (13). Furthermore, from an epidemiological point of view, subclinical cases might ensure easier spread of these protozoa. Therefore, screening pigeons for *Trichomonas* spp. should become an integral part of veterinary practice.

In this study, *T. gallinae* was detected in all four columbiform bird species examined. The infection rate was 73% which is similar to the 74% overall prevalence reported in wild columbids from western and southern Europe (9). Within the Mediterranean region, in the Iberian Peninsula 44.8% of wild and domestic pigeons were shown to carry *T. gallinae* (13), but in another study from Spain the prevalence was much higher (79.4%) (16). Furthermore, in the UK other columbiform species including *C. palumbus* and *S. decaocto* were also examined and a 60% incidence was reported (17). In Germany, four species (*C. livia, C. oenas, C. palumbus*, and *S. decaocto*) were screened and 50% of the birds were infected with *T. gallinae* (18). The prevalence was 37% among racing pigeons in Poland (10).

In Hungary, based on our screening assay, racing feral pigeons had a significantly higher prevalence of *T. gallinae* infection than urban feral pigeons (95 vs. 33%). The possible reason for this difference might be that at trading-breeding places birds of different origin are housed close to each other, and the chances for infection are higher if naive racing pigeons can get into contact with carrier birds not only outside, but inside such enclosures, e.g., by sharing food and drinking water or by kissing. Therefore, we propose that similar places play a crucial role as hotspots in the transmission of *T. gallinae*. To our knowledge, there is no similar study in Europe that compares *T. gallinae* according to the place where pigeons are kept.

All four examined wood pigeons were infected. Although the sample size was limited, this apparently high (100%) infection rate is similar to what was reported among wood pigeons in Germany (70%) (9) and the Iberian Peninsula (83.3%) (19). This high prevalence is likely associated with urbanization of wood pigeons in Hungary, resulting in close contact of birds (e.g., *via* sharing common drinking sources) in green areas where the number of wood pigeons has recently increased significantly (20).

Concerning other studied bird species, the prevalence was also high (94%) among ring doves in Hungary. There is a lack of literature data on trichomonosis of both collared dove and ring dove in Europe, despite the fact that ring doves were found to be susceptible to *T. gallinae* during experimental infection (21). In the Caribbean an outbreak was reported (22), drawing the attention of veterinarians to the necessity to monitor *T. gallinae* in this bird species.

Based on the long fragment of the 18S rRNA gene, six *Trichomonas* subtypes were detected in columbiform birds in Hungary and Romania. Compared to a reference sequence, they had up to eight nucleotide differences, meaning that the maximum genetic difference was low (0.5%) compared to what was reported from North America (3.4%) (8) and even from Austria, a country neighboring Hungary (2.9%) (23). In Hungary, two genetic variants were detected among urban feral pigeons, vs. four 18S rRNA gene subtypes occurred in racing feral pigeons kept in the same trading-breeding place, highlighting the epidemiological importance of similar facilities in general.

It is noteworthy that each bird from which *T. gallinae* was sequenced, carried a single 18S rRNA gene subtype, as also demonstrated repeatedly with different modes of detection (Supplementary Table 1, 8th column: samples TG1-50). Since it was reported that *T. gallinae* triggers premunition (24) and immunity lasts until the loss of infection (1), the *a priori* presence of any variant probably protected the relevant birds during a heterologous challenge, which is likely to occur in the environment of a pigeon trading-breeding place. It is also highly relevant to note that among racing pigeons, 18S rRNA gene subtype D was only found in pigeons that originated from Germany. We suspect that subtype D was already present in these birds when imported to Hungary and probably premunition protected them from becoming infected in the pigeon trading place by other variants.

The clinical signs relevant to trichomonosis were only associated with 18S rRNA gene subtype D in Hungary. There are several reports indicating that certain haplotypes are highly correlated with more severe lesions in various bird species (13, 16, 25). However, no close correlation has been found in terms of pathogenicity and geographical distribution when columbids were examined across Europe (9).

Considering the results in a phylogenetic context, the topology of the 18S rRNA phylogenetic tree did not show clear clustering of *T. gallinae* subtypes from this study according to host species, living place or keeping mode of columbiform birds. Clustering of *Trichomonas* sp. Hu-TG37 to the phylogenetic group of *T. canistomae* and *T. tenax* suggests that it might represent a new species. This separate position of *Trichomonas* sp. Hu-TG37 was confirmed also by the alpha-tubulin phylogenetic tree. It is known that some *Trichomonas* spp. show high genetic diversity depending on bird species, and few of these infections are caused by variants/species closely related to *T. vaginalis, T. tenax* (8, 23) or *T. canistomae* (16). *Trichomonas* sp. Hu-TG37 identified for the first time in the present study belongs to the phylogenetic group of *Trichomonas* spp. infecting (among the others) domestic carnivores. This calls for further epidemiological studies on the possible contact between dogs and pigeons (e.g., *via* water deposited in gardens in drinking bowls) and its role in the transmission of these protozoan parasites.

In conclusion, this is the first report in Hungary and Romania on the prevalence and 18S rRNA gene subtypes of *T. gallinae* in various columbiform birds using molecular methods. The results suggest that most of these variants are not host-specific and do not cause clinical signs. The highest degree of genetic diversity and high prevalence of infection was observed among racing pigeons and captive ring doves, thereby highlighting the epidemiological importance of pigeon/dove trading-breeding places.

## Data availability statement

The original contributions presented in the study are included in the article/Supplementary material, further inquiries can be directed to the corresponding author.

## Ethics statement

Ethical review and approval was not required for the animal study because all columbiform birds in this study were handled and sampled during regular veterinary care, therefore no ethical permission was needed. Written informed consent was obtained from the owners for the participation of their animals in this study.

## Author contributions

BT-S: study design, DNA extraction, data analysis, and manuscript writing. GS, AS, ÁP, and KB: sample collection. NT: PCR tests and sequencing. JK: phylogenetic analyses. ÁK and ÁJ: contribution to sample collection. JV: supervision and contribution to molecular analyses. SH: conceptualization, study design, primer design, GenBank processing, and manuscript writing. All authors contributed to the article and approved the submitted version.

## Supplementary material

Supplementary Figure 1 Lesions associated with trichomonosis in two racing pigeons: **(A)** yellowish debris in the corner of the beaks and close to the eye; **(B)** small necrotic-inflammatory foci on the palate.
