# Supplementary material for: Molecular epidemiological study of Trichomonas gallinae focusing on central and southeastern Europe
Source: Front Vet Sci. 2022 Dec 15;9:1050561. doi: 10.3389/fvets.2022.1050561 (PMC9798426; doi:10.3389/fvets.2022.1050561)

Supplementary Figure 1. Lesions associated with trichomonosis in two racing pigeons: (A) yellowish debris in the corner of the beaks and close to the eye; (B) small necrotic-inflammatory foci on the palate.

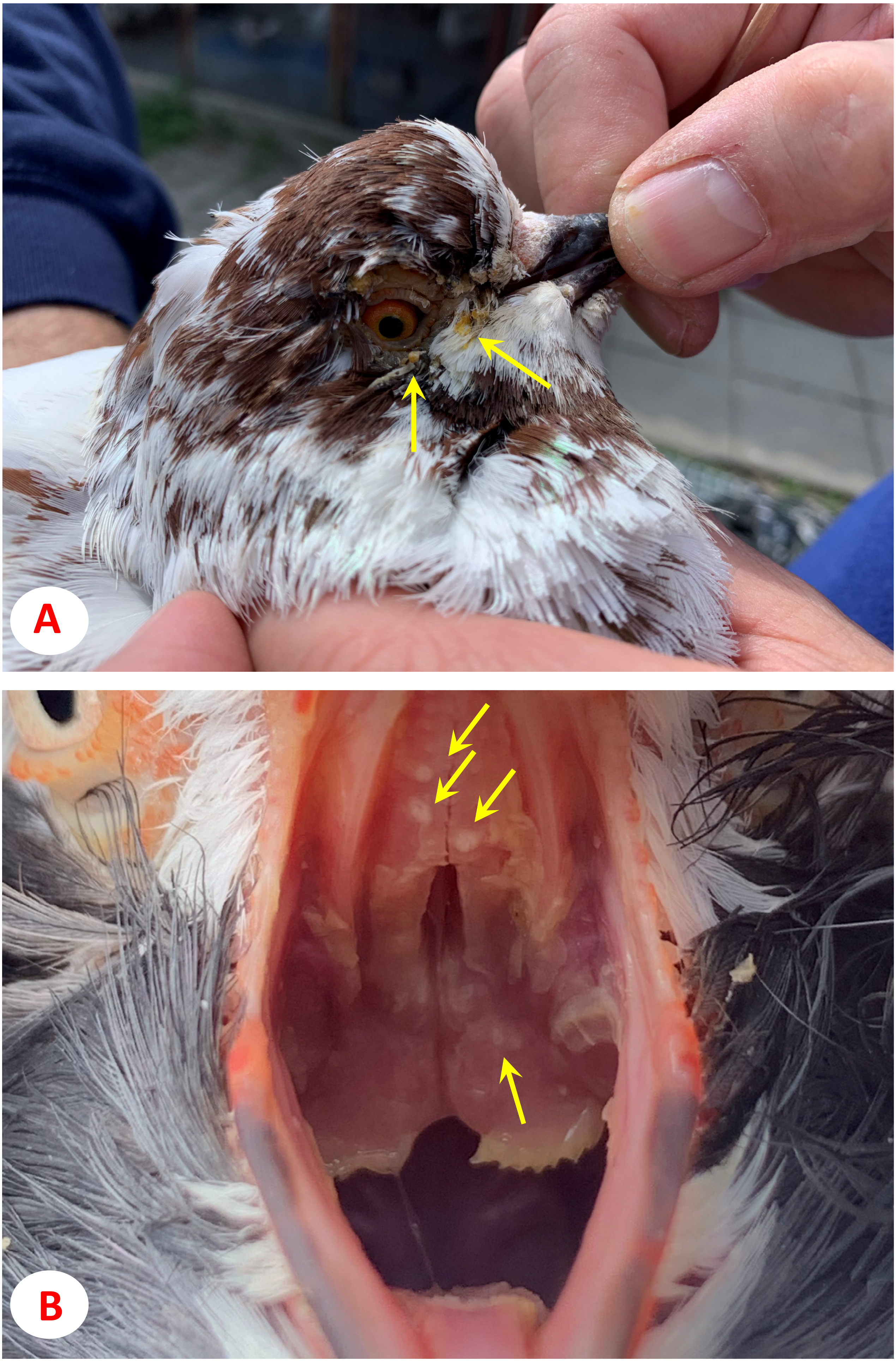

Supplement: Supplementary Figure 1 — Lesions associated with trichomonosis in two racing pigeons: (A) yellowish debris in the corner of the beaks and close to the eye; (B) small necrotic-inflammatory foci on the palate. [file Image_1.pdf]
