# Supplementary material for: Molecular epidemiological study of Trichomonas gallinae focusing on central and southeastern Europe
Source: Front Vet Sci. 2022 Dec 15;9:1050561. doi: 10.3389/fvets.2022.1050561 (PMC9798426; doi:10.3389/fvets.2022.1050561)
Supplement: Supplementary file 2 [file Data_Sheet_1.pdf]

Technical Appendix

Target genes, primers, amplicon lengths and cycling conditions of conventional PCRs used for the detection of *Trichomonas* species in this study.

PCR type	Target gene	Primer name	Primer sequence (5'-3')	Estimated amplicon length (bp)	Cycling conditions	References
**Screening assay** for short ssu fragment	ssu rDNA	**1055F**<br>**16SR1**	GGT GGT GCA TGG CCG<br>TCA CCT ACC GTT ACC TTG	500	95 °C for 5 min; 40× (95 °C for 45 s; 50 °C for 45 s; 72 °C for 1,5 min); 72 °C for 10 min;	Cepicka et al., 2006
**Primary assay** for sequencing & phylogenetic analyses	ssu rDNA (seminested PCR)	**16SL** (outer)<br>**16SR1** (outer)	TAC TTG GTT GAT CCT GCC<br>TCA CCT ACC GTT ACC TTG	1550	95 °C for 5 min; 45× (95 °C for 45 s; 48 °C for 45 s; 72 °C for 1,5 min); 72 °C for 10 min;	Cepicka et al., 2005
		**16SL** (nested)<br>**1385R** (nested)	TAC TTG GTT GAT CCT GCC<br>GAT CCT AAC ATT GTA GC	1450	95 °C for 5 min; 45× (95 °C for 45 s; 42 °C for 45 s; 72 °C for 1,5 min); 72 °C for 10 min;	
**Secondary assay** for sequencing & phylogenetic analyses	alfa-tubulin (nested PCR)	**AtubA** (outer)<br>**AtubB** (outer)	RGT NGG NAA YGC NTG YTG GGA<br>CCA TNC CYT CNC CNA CRT ACC A	1200	95 °C for 5 min; 40× (95 °C for 45 s; 55 °C for 45 s; 72 °C for 1,5 min); 72 °C for 7 min;	Edgecomb et al., 2001; Gerhold et al., 2008
		**AtubF1** (nested)<br>**AtubR1**(nested)	TAY TGY YWN GAR CAY GGN AT<br>ACR AAN GCN CGY TTN GMR WAC AT	1200	95 °C for 5 min; 40× (95 °C for 45 s; 45 °C for 45 s; 72 °C for 1,5 min); 72 °C for 7 min	

References

Cepicka I, Kutisová K, Tachezy J, Kulda J, Flegr J. Cryptic species within the *Tetratrichomonas gallinarum* species complex revealed by molecular polymorphism. Vet Parasitol. 2005;128:11–21. doi: 10.1016/j.vetpar.2004.11.003.

Cepicka I, Hampl V, Kulda J, Flegr J. New evolutionary lineages, unexpected diversity, and host specificity in the parabasalid genus *Tetratrichomonas*. Mol Phylogenet Evol. 2006;39:542–51. doi: 10.1016/j.ympev.2006.01.005.

Edgcomb VP, Roger AJ, Simpson AG, Kysela DT, Sogin ML. Evolutionary relationships among "jakobid" flagellates as indicated by alpha- and beta-tubulin phylogenies. Mol Biol Evol. 2001;18:514–22. doi: 10.1093/oxfordjournals.molbev.a003830.

Gerhold RW, Yabsley MJ, Smith AJ, Ostergaard E, Mannan W, Cann JD, Fischer JR. Molecular characterization of the *Trichomonas gallinae* morphologic complex in the United States. J Parasitol. 2008;94:1335–41. doi: 10.1645/GE-1585.1.
