# Supplementary material for: Molecular epidemiological study of Trichomonas gallinae focusing on central and southeastern Europe
Source: Front Vet Sci. 2022 Dec 15;9:1050561. doi: 10.3389/fvets.2022.1050561 (PMC9798426; doi:10.3389/fvets.2022.1050561)
Supplement: Supplementary file 3 [file Data_Sheet_2.pdf]

Supplementary Table 1. Data of sampled birds and detected *Trichomonas* 18S rRNA and alpha-tubulin gene subtypes. In the first column, slash (/) mark separates DNA sample identification (ID) number, if DNA was extracted from the same bird sampled on the same occasion 2 times (ID number of DNA from culture / ID number of DNA from swab) or 3 times (ID number of DNA from culture / ID number of DNA repeatedly extracted from culture / ID number of DNA from swab). In the second column, the year of hatching is shown whenever it was known. In the third column, the country, city (location) of sampling indicates first the pigeon breeding-trading place (Hungary, Budapest, Csepel), followed below by all other places of sampling. The fourth column is relevant only for captive birds: in their case the country of origin is shown, where the birds came from, and in parentheses the location of breeding if known. In the fifth column, the breed or habitat type, as well as any clinical signs in parentheses are shown with abbreviations explained below the table. In the sixth column, the Latin names of bird species are shown. In the seventh and eighth columns, slash (/) mark separates results of screening PCR from culture medium (marked as positive if one or both of the duplicates yielded PCR product) and swab sample, as well as the 18S rRNA gene subtype from culture medium (one or two) and swab samples. In the ninth column the designation of alpha-tubulin gene subtype is based on the corresponding 18S rRNA gene subtype, because if the latter was the same, the alpha-tubulin subtype was also consistently the same (subtypes B and C).

I.	II.	III.	IV.	V.	VI.	VII.	VIII.	IX.
**Sample ID (TG...)**	**Year of hatching**	**Country, city (location) of sampling**	**Country of origin (location of breeding)**	**Breed or habitat type (lesion)**	**Species**	**Result of screening PCR**	**18S rRNA gene subtype (acc. number)**	**Alpha-tubulin gene subtype (acc. number)**
1/11/31	2017	HU, Budapest (Csepel)	HU (Érsekcsanád)	RAP	*Columba livia*	+/+	B/B (ON631556)	NA
2/12/32	2014	HU, Budapest (Csepel)	HU (Öttevény)	RAP	*Columba livia*	+/+	B/B/B (ON631556)	NA
3/13/33	2016	HU, Budapest (Csepel)	HU (Győr)	RAP	*Columba livia*	+/+	B (ON631556)	NA
4/14/34	2020	HU, Budapest (Csepel)	HU (Budapest)	RAP	*Columba livia*	–/+	NA	NA
5/15/35	2018	HU, Budapest (Csepel)	HU (Érsekcsanád)	RAP	*Columba livia*	+/–	NA	NA
6/16/36	2012	HU, Budapest (Csepel)	HU (Vaskút)	RAP	*Columba livia*	+/+	B/B (ON631556)	B (ON808546)
7/18/38	2020	HU, Budapest (Csepel)	HU (Gara)	RAP	*Columba livia*	+/+	B (ON631556)	NA
8/19/39	2021	HU, Budapest (Csepel)	HU (Budapest)	RAP	*Columba livia*	+/+	B (ON631556)	NA
9/20/40	2021	HU, Budapest (Csepel)	HU (Budapest)	RAP	*Columba livia*	+/+	C/C (ON631557)	C (ON808547)
10/21/41	2021	HU, Budapest (Csepel)	HU (Budapest)	RAP	*Columba livia*	+/+	C/C/C (ON631557)	C (ON808547)
17/37	2020	HU, Budapest (Csepel)	HU (Gara)	RAP	*Columba livia*	+/+	TG37 (ON631566)	TG37 (ON808550)
22/42	2017	HU, Budapest (Csepel)	HU(Hajdúböszörmény)	RAP	*Columba livia*	+/+	NA	NA

23/43	2017	HU, Budapest (Csepel)	HU (Pécs)	RAP	*Columba livia*	+/+	B (ON631556)	NA
24/44	2019	HU, Budapest (Csepel)	HU (Nyőgér)	RAP	*Columba livia*	+/+	B/B (ON631556)	B (ON808546)
25/45	2016	HU, Budapest (Csepel)	HU (Felgyő)	RAP	*Columba livia*	+/+	NA	NA
26/46	2019	HU, Budapest (Csepel)	HU (Kaszaper)	RAP	*Columba livia*	–/+	B (ON631556)	NA
27/47	2020	HU, Budapest (Csepel)	HU (unknown)	RAP	*Columba livia*	+/+	C/C (ON631557)	C (ON808547)
28/48	2019	HU, Budapest (Csepel)	HU (Székesfehérvár)	RAP	*Columba livia*	+/+	B/B (ON631556)	NA
29/49	2018	HU, Budapest (Csepel)	HU (Veszprém)	RAP	*Columba livia*	+/+	B (ON631556)	NA
30/50	2018	HU, Budapest (Csepel)	HU (Öttevény)	RAP	*Columba livia*	+/+	NA	NA
51	2020	HU, Budapest (Csepel)	HU (Tiszakeszi)	RAP	*Columba livia*	+	B (ON631556)	NA
52	2018	HU, Budapest (Csepel)	Denmark	RAP	*Columba livia*	+	NA	NA
53	2016	HU, Budapest (Csepel)	Denmark	RAP	*Columba livia*	+	B (ON631556)	NA
54	2020	HU, Budapest (Csepel)	HU (Tiszakeszi)	RAP	*Columba livia*	+	NA	NA
55	NA	HU, Budapest (Csepel)	HU (Mezőtúr)	RAP	*Columba livia*	+	B (ON631556)	NA
56	2020	HU, Budapest (Csepel)	HU (Debrecen)	RAP	*Columba livia*	+	NA	NA
57	2020	HU, Budapest (Csepel)	Germany	RAP	*Columba livia*	+	D (ON631558)	NA
58	2020	HU, Budapest (Csepel)	Germany	RAP	*Columba livia*	+	D (ON631558)	NA
59	2020	HU, Budapest (Csepel)	Germany	RAP (C)	*Columba livia*	+	D (ON631558)	NA
60	2020	HU, Budapest (Csepel)	Germany	RAP	*Columba livia*	+	NA	NA
61	NA	HU, Budapest (Csepel)	Germany	RAP (E)	*Columba livia*	+	NA	NA
62	2019	HU, Budapest (Csepel)	HU (Kecskemét)	RAP	*Columba livia*	+	NA	NA
63	NA	HU, Budapest (Csepel)	HU (Kecskemét)	RAP	*Columba livia*	+	B (ON631556)	NA
64	NA	HU, Budapest (Csepel)	HU (Kecskemét)	RAP	*Columba livia*	+	B (ON631556)	NA
65	NA	HU, Budapest (Csepel)	HU (Kecskemét)	RAP	*Columba livia*	+	NA	NA

66	NA	HU, Budapest (Csepel)	HU (Kecskemét)	RAP	*Columba livia*	+	B (ON631556)	NA
67	2019	HU, Budapest (Csepel)	Germany	RAP (PM)	*Columba livia*	–	NA	NA
68	2020	HU, Budapest (Csepel)	Germany	RAP	*Columba livia*	+	NA	NA
69	2019	HU, Budapest (Csepel)	Germany	RAP	*Columba livia*	+	NA	NA
71	2019	HU, Budapest (Csepel)	Germany	RAP	*Columba livia*	+	NA	NA
72	2020	HU, Budapest (Csepel)	Belgium	RAP	*Columba livia*	+	B (ON631556)	NA
73	2021	HU, Budapest (Csepel)	HU	RAP	*Columba livia*	–	NA	NA
74	NA	HU, Budapest (Keleti)	NA	UP	*Columba livia*	–	NA	NA
75	NA	HU, Budapest (Keleti)	NA	UP (P)	*Columba livia*	–	NA	NA
76	NA	HU, Budapest (Keleti)	NA	UP	*Columba livia*	+	NA	NA
77	NA	HU, Budapest (Keleti)	NA	UP	*Columba livia*	–	NA	NA
78	NA	HU, Budapest (Keleti)	NA	UP	*Columba livia*	–	NA	NA
79	NA	HU, Budapest (Keleti)	NA	UP (P)	*Columba livia*	+	D (ON631560)	NA
80	NA	HU, Budapest (Keleti)	NA	UP	*Columba livia*	+	B (ON631559)	NA
81	NA	HU, Budapest (Keleti)	NA	UP	*Columba livia*	+	NA	NA
82	NA	HU, Budapest (Keleti)	NA	UP	*Columba livia*	–	NA	NA
83	NA	HU, Budapest (Keleti)	NA	UP	*Columba livia*	–	NA	NA
85	NA	HU, Sükösd	HU (Kecel, Dávod)		*Streptopelia risoria*	+	E (ON631565)	NA
86	NA	HU, Sükösd	HU (Kecel, Dávod)		*Streptopelia risoria*	–	NA	NA
87	NA	HU, Sükösd	HU (Kecel, Dávod)		*Streptopelia risoria*	+	NA	NA
88	NA	HU, Sükösd	HU (Kecel, Dávod)		*Streptopelia risoria*	+	NA	NA
89	NA	HU, Sükösd	HU (Kecel, Dávod)		*Streptopelia risoria*	+	NA	NA
90	NA	HU, Sükösd	HU (Kecel, Dávod)		*Streptopelia risoria*	+	E (ON631565)	E (ON808549)

91	NA	HU, Kisszállás	NA		*Columba palumbus*	+	A (ON631563)	A (ON808545)
92	NA	HU, Kisszállás	NA		*Columba palumbus*	+	NA	NA
93	NA	HU, Kisszállás	NA		*Columba palumbus*	+	NA	NA
94	NA	HU, Kisszállás	NA		*Columba palumbus*	+	NA	NA
95	NA	HU, Kisszállás	HU (Kisszállás)		*Streptopelia risoria*	+	B (ON631564)	NA
96	NA	HU, Kisszállás	HU (Kisszállás)		*Streptopelia risoria*	+	B (ON631564)	NA
97	NA	HU, Kisszállás	HU (Kisszállás)		*Streptopelia risoria*	+	NA	NA
98	NA	HU, Kisszállás	HU (Kisszállás)		*Streptopelia risoria*	+	NA	NA
99	NA	HU, Kisszállás	HU (Kisszállás)		*Streptopelia risoria*	+	NA	NA
100	NA	HU, Kisszállás	HU (Kisszállás)		*Streptopelia risoria*	+	B (ON631564)	NA
101	NA	HU, Kisszállás	HU (Kisszállás)		*Streptopelia risoria*	+	B (ON631564)	NA
102	NA	HU, Kisszállás	HU (Kisszállás)		*Streptopelia risoria*	+	NA	NA
103	NA	HU, Kisszállás	HU (Kisszállás)		*Streptopelia risoria*	+	NA	NA
104	NA	HU, Kisszállás	HU (Kisszállás)		*Streptopelia risoria*	+	NA	NA
105	NA	HU, Madaras	NA	RUP	*Columba livia*	+	NA	NA
106	NA	HU, Madaras	NA		*Streptopelia decaocto*	–	NA	NA
107	NA	HU, Madaras	NA		*Streptopelia decaocto*	+	NA	NA
109	NA	HU, Madaras	NA		*Streptopelia decaocto*	–	NA	NA
110	NA	HU, Sükösd	NA	RUP	*Columba livia*	–	NA	NA
111	NA	RO, Miercurea Ciuc	NA	UP	*Columba livia*	–	NA	NA
112	NA	RO, Miercurea Ciuc	NA	UP	*Columba livia*	–	NA	NA
113	NA	RO, Miercurea Ciuc	NA	UP	*Columba livia*	–	NA	NA
114	NA	RO, Miercurea Ciuc	NA	UP	*Columba livia*	–	NA	NA

115	NA	RO, Miercurea Ciuc	NA	UP	*Columba livia*	–	NA	NA
116	NA	RO, Miercurea Ciuc	NA	UP	*Columba livia*	+	NA	NA
117	NA	RO, Miercurea Ciuc	NA	UP	*Columba livia*	–	NA	NA
118	NA	RO, Miercurea Ciuc	NA	UP	*Columba livia*	+	NA	NA
119	NA	RO, Miercurea Ciuc	NA	UP	*Columba livia*	+	NA	NA
120	NA	RO, Miercurea Ciuc	NA	UP	*Columba livia*	+	D (ON631562)	NA
122	NA	RO, Cluj Napoca	NA	UP	*Columba livia*	–	NA	NA
123	NA	RO, Cluj Napoca	NA	UP	*Columba livia*	–	NA	NA
124	NA	RO, Cluj Napoca	NA	UP	*Columba livia*	+	NA	NA
125	NA	RO, Cluj Napoca	NA	UP	*Columba livia*	–	NA	NA
126	NA	RO, Cluj Napoca	NA	UP	*Columba livia*	–	NA	NA
127	NA	RO, Cluj Napoca	NA	UP	*Columba livia*	–	NA	NA
128	NA	RO, Cluj Napoca	NA	UP	*Columba livia*	+	A (ON631561)	NA
129	NA	RO, Cluj Napoca	NA	UP	*Columba livia*	–	NA	NA
130	NA	RO, Cluj Napoca	NA	UP	*Columba livia*	–	NA	NA
131	NA	RO, Cluj Napoca	NA	UP	*Columba livia*	+	D (ON631562)	D (ON808548)
132	NA	RO, Cluj Napoca	NA	UP	*Columba livia*	–	NA	NA
133	NA	RO, Cluj Napoca	NA	UP	*Columba livia*	–	NA	NA

Marks, abbreviations: NA = not available, Keleti = Keleti railway station, RAP = racing feral pigeon, UP = urban feral pigeon, RUP = rural feral pigeon, HU = Hungary, RO = Romania. **Lesions**: E = eye secretions without conjunctivitis, C = conjunctivitis, P = point-like lesions in the pharynx, PM = pseudomembrane in the pharynx.
